# The Information Architecture of Behavior Change Websites

**DOI:** 10.2196/jmir.7.2.e12

**Published:** 2005-05-18

**Authors:** Brian G Danaher, H Garth McKay, John R Seeley

**Affiliations:** ^1^Oregon Research InstituteEugene, ORUSA

**Keywords:** Health behavior, Internet, behavioral research, information architecture, cigarette smoking, tobacco

## Abstract

The extraordinary growth in Internet use offers researchers important new opportunities to identify and test new ways to deliver effective behavior change programs. The information architecture (IA)—the structure of website information—is an important but often overlooked factor to consider when adapting behavioral strategies developed in office-based settings for Web delivery. Using examples and relevant perspectives from multiple disciplines, we describe a continuum of website IA designs ranging from a matrix design to the tunnel design. The free-form matrix IA design allows users free rein to use multiple hyperlinks to explore available content according to their idiosyncratic interests. The more directive tunnel IA design (commonly used in e-learning courses) guides users step-by-step through a series of Web pages that are arranged in a particular order to improve the chances of achieving a goal that is measurable and consistent. Other IA designs are also discussed, including hierarchical IA and hybrid IA designs. In the hierarchical IA design, program content is arranged in a top-down manner, which helps the user find content of interest. The more complex hybrid IA design incorporates some combination of components that use matrix, tunnel, and/or hierarchical IA designs. Each of these IA designs is discussed in terms of usability, participant engagement, and program tailoring, as well as how they might best be matched with different behavior change goals (using Web-based smoking cessation interventions as examples). Our presentation underscores the role of considering and clearly reporting the use of IA designs when creating effective Web-based interventions. We also encourage the adoption of a multidisciplinary perspective as we move towards a more mature view of Internet intervention research.

## Information Architecture Designs

Attracted by the Internet's tremendous reach, its economies of scale, as well as its ability to foster instantaneous interaction and individual tailoring, behavioral science and health care researchers are beginning to port their individual and group-based interventions to the Internet in increasing numbers [1,2]. These researchers are finding, however, that this translational process is not simple since they are faced with a new set of challenges inherent in adapting their content and interventions to take fuller advantage of the unique capacities of the Internet to encourage measurable behavior change. One of the critical dimensions worthy of greater scrutiny is a website's information architecture (IA), which Garrett defines as the structure of information space to facilitate intuitive access to content and task completion [3]. For example, how much of an Internet-based behavior change intervention's success—or lack thereof—is due to the format, presentation, and quality of the website's IA apart from the soundness of the underlying theory and substance of the intervention? While the literature is currently lacking on this issue, a logical place to start is to examine common types of website IA and how these designs might best support behavior change processes.

We acknowledge the important role played by reviews that attempt to rate the adequacy of behavior change websites [4-10]. However, we believe that the promise of using the Internet as a delivery channel or modality for behavior change programs also warrants parametric research that focuses on the interaction between website IA features and the requirements of successful behavior change [11].

## Website Design Elements

**Figure 1:**
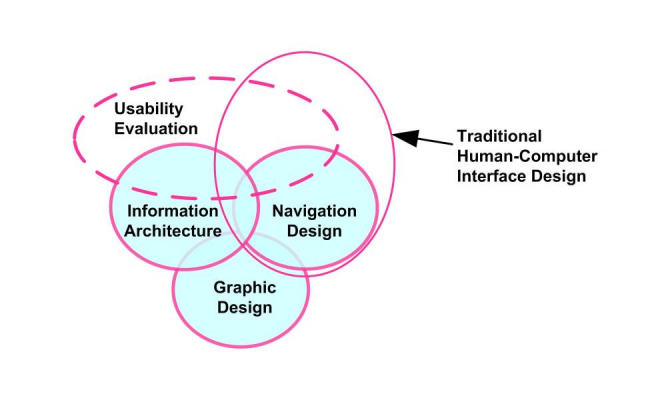
Website design elements (used with permission [17])

Rapidly emerging design principles that take into consideration current practices as well as empirical data that describe how users best interact with website content can serve as new guides to the design and information structuring of websites [12,13]. As website conventions become more widely adopted, users will be able to navigate websites successfully without having to process the underlying structural and usability “rules” in a conscious manner [14-17]. Yet standardization will undoubtedly be a difficult goal to achieve in any final form since new website designs and browser capabilities that try to escape the limitations of today's browser experience inevitably emerge (see Garrett's discussion of Ajax [18]). As depicted in Figure 1, multiple disciplines contribute to the overall design of any website, including graphic design (the visual and aesthetic communication of information), navigation design (methods to help users find their way around a website), and IA (the coherent structure and display of content) [17].

Users of most websites typically enjoy considerable freedom when it comes to accessing content. For example, they can choose when they want to visit the site, what they want to browse, how much they want to see, how much time they want to spend seeing it, in what order their browsing will occur, what else they might be doing/viewing or listening to while browsing, and whether they want to copy, save, and/or print content as they review it. Some researchers have postulated that this type of unrestricted (*ad lib*) interaction between users and websites shares meaningful similarities with the manner in which wild animals forage for food. From this *information foraging* perspective, users are free to follow the “information scent,” which helps them determine if the effort of the search will be rewarded by finding desired nuggets or *chunks* of information [16,19-21]. “Novice users…perform a kind of *hill-climbing* with information scent as the heuristic for choosing the next step to take” [16].

In this report, we describe IA structures that appear to have particular relevance for websites intended to help users change their health behavior. In particular, we focus on four IA designs: (1) the free-form *matrix* design that offers little information structure, (2) a *hierarchical* design that provides the user with information arranged in an organized fashion, (3) a *tunnel* design that defines a narrow path with a predefined series of steps, and (4) a *hybrid* design composed of a combination of modules that have their own IA design.

## Matrix Design

**Figure 2:**
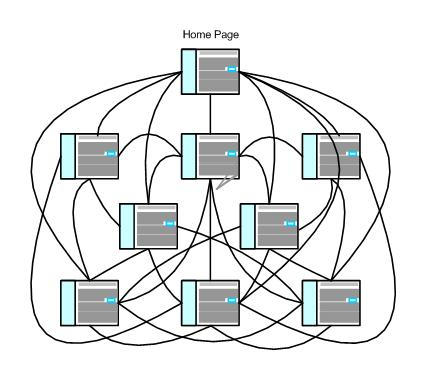
Matrix design schematic

Websites with a matrix IA design embody the principles of the originators of hypertext, HTML, and the Web [22,23], and they take fullest advantage of HTML's hyperlink capabilities to allow users to review all website content (Figure 2). (Note that the lines in Figure 2 that connect Web page icons represent the multiple links that enable users to move from one Web page to another.) In the matrix IA design, users are free to pursue their idiosyncratic interests by using their own path through the available content. When properly created, this design can expedite a user's search of the content. When links are too numerous or do not anticipate a user's search pattern, then the user may well have to search through all available listings. Examples of the matrix design can be readily found in government sponsored websites focused on broad health topics.

### Rationale for use

The matrix design can be very efficient in that it offers the user the maximum amount of content within the confines of a Web page, and it uses multiple links that transport the user to content available on many different pages. It is particularly well-suited for finding information although its efficiency is associated with how well the links anticipate the user's search preferences. Moreover, the freedom of movement and exploration associated with the matrix design may come at a cost because users may become disoriented, quite literally *lost in hypertext,* and may experience great difficulty when trying to retrace their steps to review what they have already seen [24]. As a result, Lynch and Horton [12,25] have suggested that a website with a matrix design may not be well-suited to helping users become familiar with a new content area. Instead, they recommend that the matrix design is most applicable to small websites that are designed for use by highly educated and experienced users who are already familiar with the basic organization of the content and who are visiting in order to obtain further education or enrichment.

## Hierarchical Design

**Figure 3:**
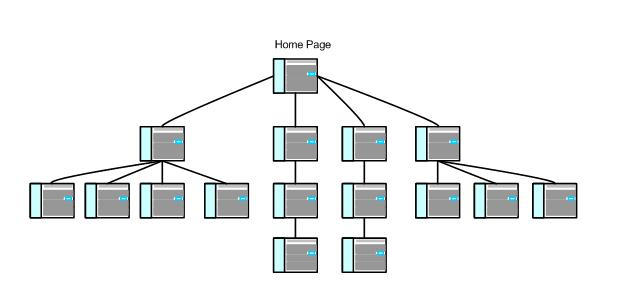
Hierarchical design schematic

In hierarchical IA designs, information is organized in a *top-down* manner so that the user can review increasingly detailed content. The user is presented with small *chunks* of information that he/she can rapidly explore in a nonsequential manner. The design depicted in Figure 3 contains three instances of a *one-to-many relationship* in which a single Web page contains links to the home page and two second-level pages. In contrast to the matrix design (Figure 2), the hierarchical design has significantly fewer links between pages [12,25]. Hierarchical IA designs help users find desired content by locating a broad theme and then *drilling down* into more detailed information. And it is relatively easy to find your way back through content already viewed because it simply involves moving back up the hierarchical structure.

### Rationale for use

Websites with a hierarchical design tend to reduce the kind of confusion that comes from users being presented with too many links and options (as may be associated with the matrix design). In addition, many users are familiar with information arranged in hierarchical fashion since it is similar to a table of contents design and it mimics the tree-like file directory/subdirectory structure that operating systems use to organize files [26]. Businesses often use hierarchical models to organize information, workgroups, project plans, etc [12,26]. The usefulness of a hierarchical design diminishes if the content is nested too deeply (in too many levels). When this occurs, the burden on the user is increased because of the added effort required to drill down through so much content in order to locate the desired information [21]. In addition, a user may become confused unless his/her mental model of the content grouping, and even the labels used to describe the content groups, corresponds to the way that content is organized on the website [16,20,25].

## Tunnel Design

**Figure 4:**
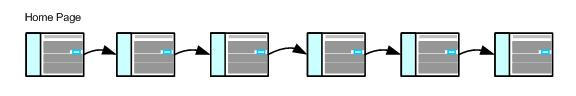
Tunnel design schematic

Websites based on a *tunnel* IA design represent the opposite end of the continuum from a matrix design. Instead of free access to content, the user follows a step-by-step (page-by-page) approach (Figure 4). This design eliminates access to any ancillary or related Web pages that are viewed as potential distractions.

An example of task-based tunnel design is encountered when purchasing items on the Internet. For example, when purchasing travel tickets online, the user typically follows a sequence of steps, each having its own Web page that shows the following: (1) day and time of flight choices, (2) hotel and car rental details, (3) credit card information, (4) purchase confirmation, and (5) booked reservation details. Note that van Duyne et al [17] refer to this online purchasing scenario as a *process funnel*. Another common use of the tunnel design can be found in online surveys [27].

While emerging website design conventions take into consideration matrix or hierarchical designs, there is relatively little agreement on how best to use tunnel IA designs. The structure of many websites with tunnel IA designs seems to have been derived from the instructional designs found in corporate multimedia CD-ROMs. Almost all e-learning courses adhere to a tunnel design. These typically have a series of lessons that present the content, test for comprehension, and provide remedial loops and other conditional branching [28].

It should be noted that the tunnel IA design presents significant challenges since HTML was designed as hypertext markup for documents rather than a software interface for Web applications. Indeed, creating a tunnel essentially requires the designer to break the rules of the hypertext and the Web in order to guide the user's experience, as is clearly indicated in the guidelines that reviewers of tunnel IA designs have recommended [12,15,17]:

- Display extra information in pop-up windows instead of the browser in order to reduce the possibility that users will leave the tunnel.
- Remove all standard browser tools, including navigation bars, tab rows, location breadcrumbs, and embedded links.
- Limit navigation to “next” and “prior” buttons.
- Provide a progress bar to show users the context of where they are in the process.
- Make it clear how to proceed to the next step.
- Include error messages at the time the errors occur.

Little is currently known about how users accommodate the unfamiliar confines of a website based on a tunnel IA design. Nielsen, a noted Web usability authority, has argued that “…one of the Web's most powerful features is that it lets users control their own destiny. Users go where they want, when they want.... Websites that force users to sit through sequences with nothing to do will be boring and pacifying, regardless of how cool they look” [29].

The challenge may well be to design tunnel websites that encourage users to be patient long enough to become comfortable using an unfamiliar program interface that is designed to keep them from engaging in their typical information foraging behavior. Some may find this to be a frustrating experience. Users who are matriculating through an e-learning program (eg, students, employees receiving online training or obtaining career critical certification) may be more motivated to cope with the frustration and accept the constraints of tunnel designs than would most prospective participants of Web-based behavior change programs. Other users may greatly value the reduced complexity that the tunnel provides, avoiding the information anxiety that can accompany a program that offers a myriad of links and options from which to choose [30,31].

### Rationale for use

There are a number of arguments in favor of designing websites with a tunnel format. The linear model is familiar because it is consistent with the manner in which content is presented in movies [28,32], textbook narratives [26], academic classes, and multiple clinical sessions. Its use assumes that there is some optimal ordering and/or *dosage* of content that is associated with greater effectiveness. In contrast, a matrix design website affords little control over the order and amount of content actually reviewed.

The tunnel IA design is particularly well-suited to fostering the type of *dialog* that can be associated with multi-session programs in which users are assigned tasks to do at home on their own in between online sessions. At the start of a subsequent session, users can be asked about any problems and the progress they experienced during the practice of these tasks. This dialog sets the stage for the program to provide tailored feedback and recommendations. In addition, programs using a tunnel IA design can more carefully titrate the amount of information a user is exposed to in order to reduce the sheer number of strategies and the amount of program content that the user learns and potentially uses.

Finally, it is important to acknowledge that tunnel programs are not, by definition, inflexible. For example, they can be targeted in the sense that content in the tunnel can be adapted to better address a particular demographic audience. They can also be tailored in the sense that the program can contain tests of knowledge as well as comprehension of key learning points, along with remedial loops as necessary.

## Hybrid Designs

**Figure 5:**
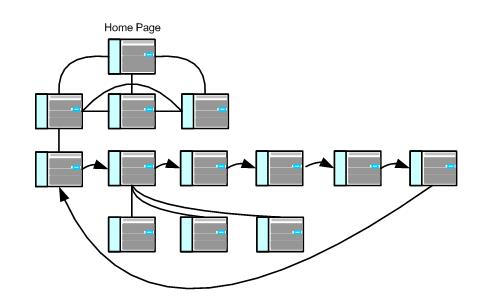
Hybrid design schematic #1

Hybrid designs are composed of multiple IA *modules,* each of which can be described along the continuum from matrix and tunnel designs. It is possible to mix and match matrix, tunnel, and hierarchical designs. For example, the hybrid design depicted in Figure 5 uses a tunnel design combined with a module that adheres to a hierarchical IA design that offers users optional, but clearly defined, content while moving along the required sequence of steps. Note in Figure 5 that the user has free access to three Web pages from the home page (a matrix design). On one of these pages the user can choose to enter a program composed of a series of sequential steps (a tunnel design). On the second page of the tunnel design the user can sample from the content of any of three linked pages without interrupting the step-by-step flow of the process. This allows the user to explore content (engage in discovery learning) while still maintaining the focused forward movement of the tunnel program.

While tunnel designs require few navigational controls other than the *prior* and *next* buttons, ancillary Web pages may have far richer content that requires additional navigational controls (similar to those found in a matrix IA design). Changing navigational tools as users move from ancillary pages back to the sequential tunnel pages can present usability challenges. Similarly, if ancillary pages provide links to Web page resources outside of the behavior change program, some users might choose to leave the current session while others might not be able to find their way back to their point of departure [26].

It is also possible to adapt the tunnel design so that it morphs into a more flexible design once the user has completed a required step of content. When the user has seen *all* of the required content contained in a tunnel (accomplished all of the required steps in the required order), then the IA of that Web-based program can change from a tunnel to a matrix so that the user can freely access any of the available content. Note that the ease of transforming a website from a tunnel to a matrix IA design is greatly improved when the sites are not created using hand-coded HTML. Instead, these transformations require the development of carefully modularized, data-driven websites that display content based on the interaction of logic scripts (eg, PHP, ASP, ColdFusion), SQL databases, and cascading stylesheets. By capturing and interpreting user data, and then manipulating scripts, databases, and stylesheets, it is possible to adapt the appearance and behavior of websites in real time.

**Figure 6:**
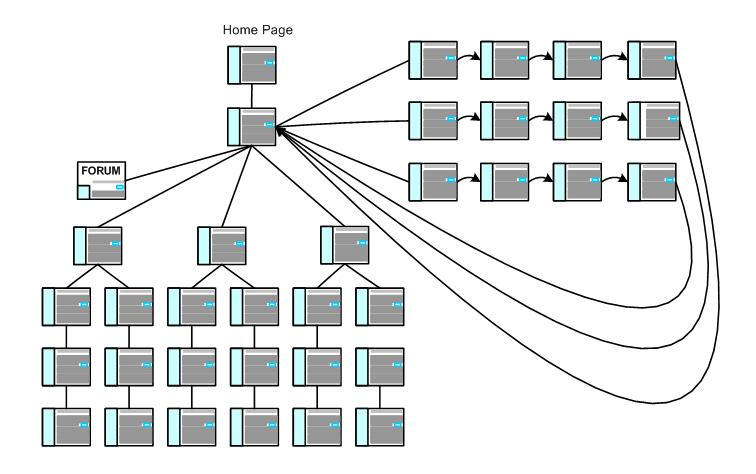
Hybrid design schematic #2

A somewhat more complex hybrid design is depicted in Figure 6. In this example, the user starts out by accessing an initial Web page that contains a welcome and log-in that enables access to a page that provides matrix-like access to seven content areas, including a Web forum, three hierarchical IA designs used to present articles of content in increasing detail, and three tunnel IA design experiences that walk the user through the content in a step-by-step manner.

### Rationale for use

Hybrid IA designs appear to have a number of distinct advantages over websites that offer only the more orthodox matrix or tunnel designs. For example, hybrid designs can give users more guidance than can be obtained from matrix IA designs. Hybrid designs also allow the user to break free from the lock-step sequence of pages found in a tunnel design. Offering alternative ways of interacting with content can be refreshing. It can spur the user to become more involved in his/her own learning rather than falling into a mode of a passive page turner. Depending upon what is contained on the ancillary Web pages, the user can have a far richer and more effective learning experience and outcome. For example, the potential impact of ancillary pages in the hybrid design could enable the user to customize his/her experience by joining a Web forum, viewing pertinent video vignettes, or reviewing more in-depth articles.

It is also important to note that hybrid designs may well reduce attrition by users who find the tunnel experience to be too constraining. No matter how efficacious a tunnel-based program is found to be, its effectiveness can be seriously undermined if users find the experience too unfamiliar, inflexible, and, thus, unpalatable.

Table 1 presents an overview of the strengths and constraints of the IA designs discussed in this paper.

**Table 1:** Summary of IA design features

**IA Design**	**Strengths**	**Constraints**
Matrix	Can move freely through content Encourages discovery learning	Links may not anticipate user's search pattern User can become disoriented
Hierarchical	Familiar top-down organization Provides a simplified view Easy to retrace steps	Deeply nested information may be difficult to find Labels may not correspond to how user defines area
Tunnel	Familiar step-by-step flow through content Can control timing and amount of exposure to content	Does not follow familiar website navigation conventions May cause frustration and reduce follow-through
Hybrid	Uses multiple IA designs that best fit content and purpose	Moving between Web pages with different IA designs may present usability challenges

## Behavior Change Examples: Tobacco Cessation

### Oregon Center for Applied Science

**Figure 7:**
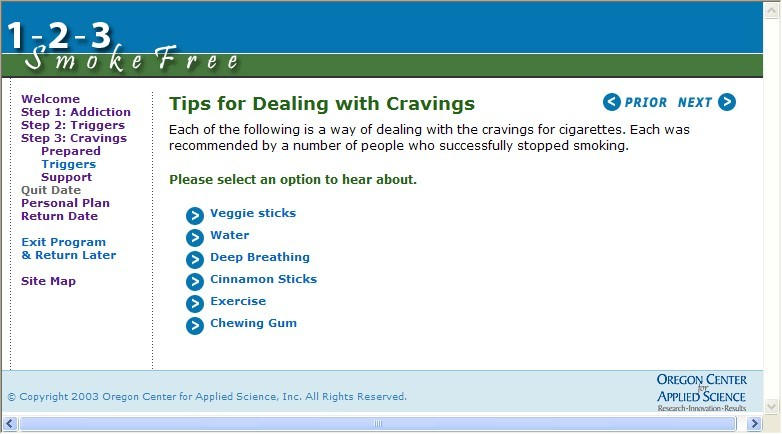
1-2-3 SmokeFree Web page showing hybrid IA design (tunnel with links to optional ancillary content)

The most recent version of the 1-2-3 SmokeFree Web-based smoking cessation program developed by Oregon Center for Applied Science [33] uses a hybrid IA design in which the user moves through an extended tunnel containing more than 20 sequential steps that address the key topics of addiction, triggers, cravings, picking a quit date, and making a personal quit plan. Eight of these Web pages are based upon a hierarchical IA design which allows the user to access additional cessation content on other pages. The screen capture of one of these Web pages depicted in Figure 7 shows how the user can either continue to move forward within the tunnel by pressing the *next* button, or, alternatively, can select any of the available links that provide additional tips for dealing with cravings. In this smoking cessation program, the user is able to reverse direction in the program (via the *prior* button or using the expand/collapse features of the left navigation bar) in order to review any of the content already covered. In keeping with the tunnel design, however, the user is encouraged to move forward to work with new content in a required order.

### National Cancer Institute

In another example, a National Cancer Institute (NCI) website on smokeless tobacco [34] presents general information adapted from a booklet into a series of six Web pages that adhere to a tunnel IA design. The user can click to move forward or backward from one page to the next as the content is presented in a linear manner.

Finally, another NCI website (Smokefree.gov) provides an “online guide to quitting” that uses a hybrid design [35]. More specifically, the Website uses a hierarchical IA design that enables users to click on headings in a table of contents that allows them to select and then drill down to learn more about any content area in any order. Once they arrive at more detailed information on deeper Web pages, users see links that allow them to break out of the hierarchy and leapfrog into another broad topic area using a variation on the tunnel IA design: “Move on the Preparing to Quit,” “Move on to Quitting,” and then “Move on to Staying Quit.”

## Discussion

The development of effective Internet-based behavior change programs presents a number of unique challenges. It is reasonable to assume, for example, that the best practice approaches drawn from office-based settings (see, for example, [36]) will need to be adapted to fit the strengths of Web delivery. In addition, the content of behavior change interventions must be presented in a way that is attractive as well as usable in order for it to have beneficial impact.

For example, the more free-form matrix IA designs might be particularly well-suited to a website (or portion of a large website) designed to help users resolve their ambiguity regarding whether or not to engage in a behavior change attempt [37]. Perhaps participants who are more committed and *ready* to change would be best matched with a tunnel IA design that guides them through the step-by-step change (see, for example, [38]). And perhaps any tunnel design behavior change program would be improved by the addition of a module that allows users to explore what is known about the risks and benefits of making the behavior change as well as their feelings regarding the change.

The rationale for using any particular IA design is largely theoretical rather than validated or universally accepted. We anticipate a period of intriguing discussion and related empirical testing regarding the ways to take fullest advantage of Internet-based programs. Highly relevant topics abound, including websites that use different IA designs, the value of tailoring and targeting content, scheduling of homework tasks and the tracking of progress, roles of media and interactivity, structure and value of community components (eg, Web forums), impact of email and/or phone adjuncts, etc. Early examples exploring these and related research directions have already begun to emerge for different target behaviors, as in diabetes [39], eating disorders [40], post-traumatic stress (see tunnel IA design in [41]), depression [42], smoking cessation [43-46], caregiving [47], and also for tests of different program components as in formats and user preference for multimedia [48,49].

The speed with which technology is evolving is staggering. The Internet has rapidly become an accepted part of daily life for hundreds of millions of people worldwide. As a result, it is reasonable to conclude that these revolutionary advances will act as a catalyst to expand the scope and impact of both persuasive technology, in general [30,50], and of Internet-based health behavior change programs [51]. We have highlighted the important role that IA designs can have upon the design and likely impact of online behavior change programs. We believe that a broad multidisciplinary perspective is needed in order to better understand the larger context of relevant creative thinking and empirical research, to define and test both theories and strategies, and to deliver more innovative and effective Internet behavior change programs.

## Abbreviations

IA: information architecture
NCI: National Cancer Institute
